# Individual variation in food odor sensitivity is related to neuropeptide signaling and starvation in *Drosophila*

**DOI:** 10.1016/j.isci.2026.118105

**Published:** 2026-11-04

**Authors:** Misako Okumura, Yoshiaki Shibukawa, Yumiko Ukita, Akira Ito, Mami Nakamizo-Dojo, Takahiro Chihara

**Affiliations:** 1 Program of Biomedical Science, Graduate School of Integrated Sciences for Life, Hiroshima University, Higashi-Hiroshima, Hiroshima 739-8526, Japan; 2 Program of Basic Biology, Graduate School of Integrated Sciences for Life, Hiroshima University, Higashi-Hiroshima, Hiroshima 739-8526, Japan; 3 Department of Biological Science, School of Science, Hiroshima University, Higashi-Hiroshima, Hiroshima 739-8526, Japan

**Keywords:** olfactory sensitivity, individual difference, *Drosophila melanogaster*, olfactory trap assay, starvation, transcriptome

## Abstract

Olfaction is fundamental for animal survival, and individual variation exists in olfactory sensitivity. However, the underlying mechanisms of such variations remain unclear. We classified *Drosophila* with different olfactory sensitivities toward food-related odors from the same genetic stock using sequential olfactory trap assays. Transcriptome analysis of sensitive and insensitive flies, defined by consistent attraction or non-attraction across three assays, revealed differential expression of genes associated with chemosensation, lipid metabolism, and lipid transport. Flies attracted to food-related odors exhibited lower triacylglycerol levels than non-attracted flies. Sensitive flies also expressed higher levels of *sNPF-R*, a neuropeptide receptor induced by starvation, despite all flies being exposed to the same starvation stress before the trap assays. Moreover, sensitive flies were more vulnerable to starvation, and feeding decreased their attraction to food-related odors. Together, these findings indicate that individual variation in internal physiological state, particularly starvation level, influences olfactory sensitivity to food-related odors through neuropeptide signaling.

## Highlights

• Sequential trap assays identify flies with high and low food odor sensitivity
• Transcriptome analysis reveals differences in lipid-related gene expression
• Triacylglycerol levels and starvation resistance differ with odor sensitivity
• Short neuropeptide F receptor expression is associated with food odor sensitivity

## Graphical abstract

**Figure:**
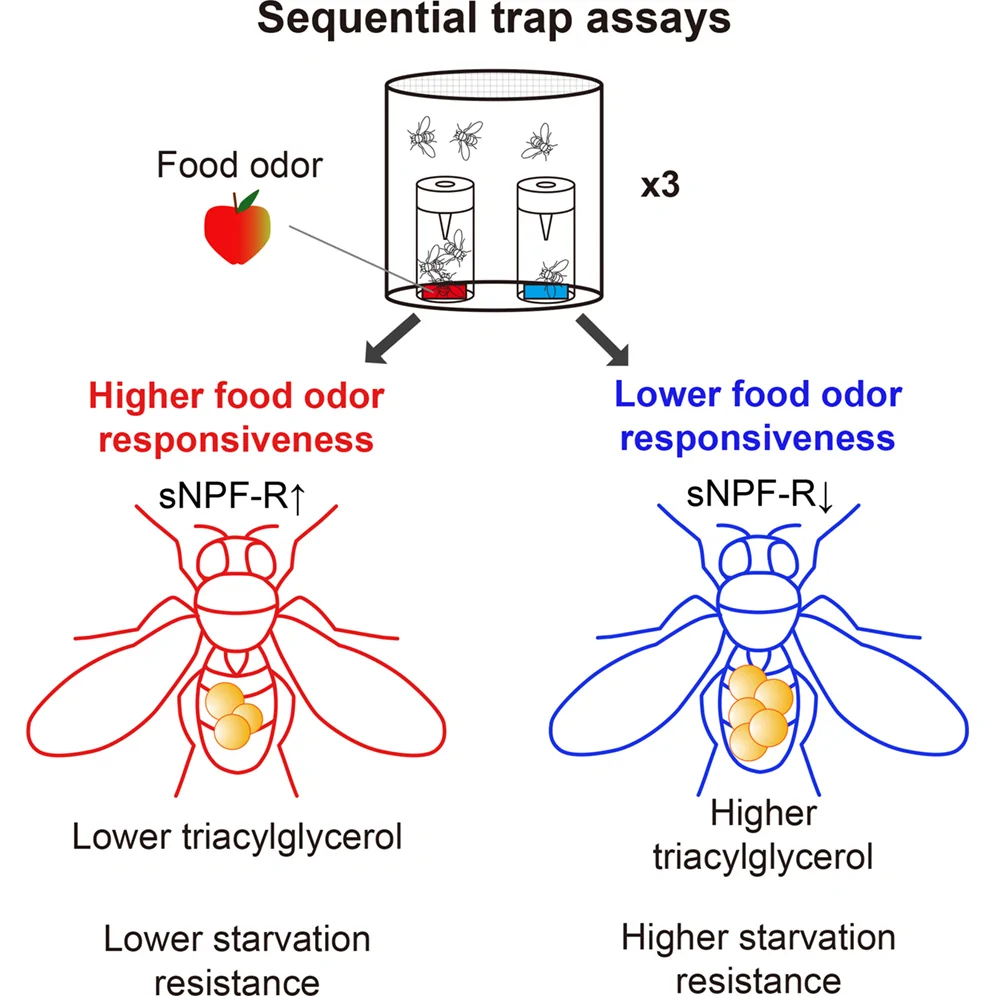

## Teaser

Animal physiology; Biological sciences; Sensory neuroscience; Transcriptomics

## Introduction

Animals possess the ability to detect environmental cues, such as food, predators, and mating partners, with the help of their olfactory system. Olfactory sensitivity varies among individuals of the same species. One cause of this variation is the genetic variation in olfactory receptors. In humans, polymorphism of the OR7D4 olfactory receptor results in different perceptions of the odorous steroid androstenone.^1^ Additionally, an individual’s physiological state can influence olfactory sensitivity. For instance, olfactory sensitivity is known to increase during pregnancy.^2^ Many animals, including humans, are more sensitive to food-related odors when they are starved than when they are satiated.^3^^,^^4^^,^^5^^,^^6^ This study investigated whether variations in olfactory sensitivity correlate with individual differences in physiological states among individuals who share the same environment and genetic background.

The olfactory system of *Drosophila melanogaster* serves as an exemplary model for studying individual variations in olfactory perception. Research has extensively explored how flies respond to specific odors, focusing on the underlying neural and molecular mechanisms, including the roles of olfactory receptors, their corresponding ligands, and the complexities of olfactory neural circuits.^7^^,^^8^^,^^9^^,^^10^^,^^11^ In addition, previous studies have shown that a highly inbred laboratory strain displays idiosyncratic odor preferences,^12^ indicating that individual variations in olfactory perception exist within this species.

Olfactory sensitivity in *Drosophila* is closely associated with its physiological state. For instance, starvation heightens sensitivity to attractive odors, such as apple cider vinegar (ACV), which is detected by multiple olfactory receptors, including Or42b.^11^^,^^13^ This enhanced sensitivity during starvation is due to the presynaptic facilitation of the Or42b neurons, which is mediated by the upregulation of sNPF-R, a short neuropeptide F receptor within these neurons.^5^ Nevertheless, it remains unclear whether variations in physiological conditions contribute to individual differences in olfactory sensitivity to food-related odors.

In this study, we conducted sequential olfactory trap assays to identify flies with varying sensitivities to ACV. We selected ACV because it is a complex and highly attractive food-related odor and provides a biologically relevant stimulus to examine individual variation in olfactory sensitivity. The flies were classified into two groups based on their responses across three sequential trap assays: flies attracted in all three assays were defined as “sensitive flies”, while flies that were not attracted in all three assays were referred to as “insensitive flies”. We examined the DNA sequence and measured the mRNA levels of the Or42b gene in both groups, as well as the number of Or42b neurons. Transcriptome analysis revealed distinct gene expression profiles between the sensitive and insensitive groups. Notably, sensitive flies exhibited higher levels of sNPF-R mRNA, implying that these flies may experience greater starvation levels than their insensitive counterparts. This suggests a correlation between sensitivity to food-related odors and individual starvation levels. Moreover, sensitive flies demonstrated increased vulnerability to starvation, and their attraction to food-related odors decreased post-feeding. Our findings indicate that individual variations in internal physiological states, such as starvation, affect olfactory sensitivity via neuropeptide signaling.

## Results

### Sequential olfactory trap assays distinguish the individual differences in sensitivity to ACV

Generally, behavioral assays are performed multiple times to examine the phenotypic tendencies caused by genetic or environmental manipulations and to find the average individual differences even in the same genetic strains. We were interested in the genetic and physiological differences that could explain individual differences, especially in olfactory sensitivity. To select flies with different olfactory sensitivities to ACV, which is an attractive odor for fruit flies, we performed olfactory trap assays sequentially (Figures 1A–1C). Each trap assay was performed for 2 h, such that the score of each assay reflected the individual olfactory sensitivity. Flies attracted to the ACV in the first assay were re-tested in the second assay, and those attracted to the ACV in the second assay were re-tested in the third assay. We examined the preference index (PI) of the Canton-S strain and found that the PIs significantly increased in the second and third assays (Figure 1D). In contrast, we repeatedly selected non-attracted flies by collecting those that remained in the field or control bottles (Figures 1A and 1C). The PIs of the non-attracted flies in the second and third assays were significantly lower than those of the attracted flies (Figure 1D).

**Figure 1:**
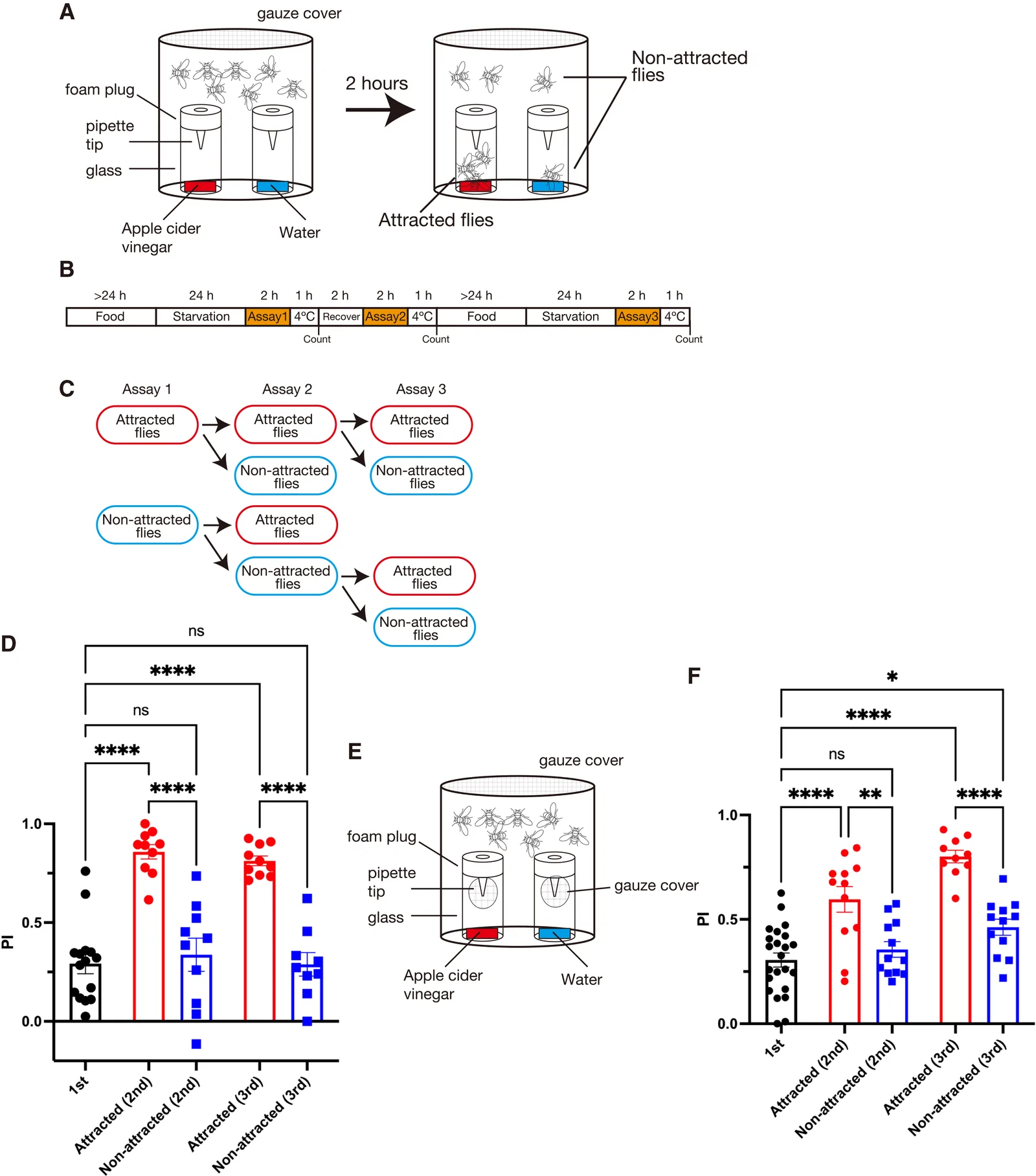
Classification of attracted and non-attracted flies using sequential trap assays (A) Schematic image of the olfactory trap assay. Approximately one hundred flies were transferred to a 1000-mL glass beaker covered with gauze. Two glass bottles with a truncated pipette tip inserted into a foam plug were placed in the beaker. Pieces of paper soaked with apple cider vinegar (ACV; red) or water (blue) were placed inside the two glass bottles. After a 2-h assay in the dark, flies inside the ACV bottle were categorized as attracted flies, and those remaining in the field or inside the water bottle were categorized as non-attracted flies. (B) Time course of the sequential trap assays: Flies were starved for 24 h before the first assay. After the first assay, they were anesthetized at 4°C for 1 h and counted, followed by a 2-h recovery period without food at 25°C before the second assay. After the second assay, the flies were again anesthetized at 4°C for 1 h and counted, then re-fed for at least 24 h and subsequently starved for 24 h before the third assay. After the third assay, the flies were anesthetized at 4°C for 1 h and counted. (C) Strategy for the sequential trap assays. Attracted and non-attracted flies recovered from each assay were separately subjected to the subsequent assay. All three assays were performed using 25% ACV. (D) Preference index (PI) in the three sequential trap assays. PIs are shown for the initial population in the first assay (black) and for the attracted (red) and non-attracted (blue) flies in the second and third assays. Data are represented as mean ± standard error of the mean (SEM). One-way analysis of variance (ANOVA) with Šidák’s multiple comparisons test; ns, not significant; *∗∗∗∗p* < 0.0001; 1st, *N* = 15; non-attracted (3rd), *N* = 9; others, *N* = 10. N represents the number of independent trap assays. (E) Schematic of the modified trap assay in which the opening of each pipette tip was covered with gauze. This allowed flies to detect the odors while preventing direct contact with or ingestion of ACV and water. (F) PIs obtained using the modified trap assay shown in (E). PIs are shown for the initial population in the first assay (black) and for the attracted (red) and non-attracted (blue) flies in the second and third assays. Data are represented as mean ± SEM. One-way ANOVA with Šidák’s multiple comparisons test; ns, not significant; *∗p* = 0.0363, *∗∗p* = 0.0021, *∗∗∗∗p* < 0.0001; 1st, *N* = 23; attracted (3rd), *N* = 10; others, *N* = 12. N represents the number of independent trap assays.

ACV can be a food source for starved flies, and it is possible that the attracted flies consume ACV, which affects the choice of the following assays. To address this possibility, the pipette tips within the trap bottles were covered with gauze so that the flies could smell the odor but could not touch or ingest the ACV and water (Figure 1E). The PIs of the attracted flies in the second and third assays were also significantly higher than those of the non-attracted flies (Figure 1F). This result suggests that the increase in PIs in the attracted flies was not due to the rewarding experience of the previous assays. These results demonstrate that within a population, some flies are sensitive to ACV, whereas others are insensitive.

### Olfactory sensitivity to ACV was not related to Or42b expression, the number of Or42b neurons, or Or42b polymorphism

To select flies that were more sensitive to ACV, we decreased the concentration of ACV in the second and third assays: 25% ACV in the first assay, 5% in the second assay, and 1% in the third assay (Figures 2A and 2B). Flies attracted in a single assay are referred to as “attracted flies”, and those not attracted are referred to as “non-attracted flies”. Flies attracted in all three consecutive assays with ACV were categorized as “sensitive flies”, whereas flies that consistently failed to respond to 25% ACV were categorized as “insensitive flies”. In the selection of sensitive flies, the PI did not differ significantly among the three sequential assays, despite the progressive reduction in ACV concentration from 25% to 5% and 1% (Figure 2C). Similarly, no significant differences in PI were detected among the three assays used to select insensitive flies with 25% ACV (Figure 2D). These results suggest that this sequential selection procedure enabled us to collect relatively high-sensitivity flies and flies that consistently showed low responsiveness to ACV.

**Figure 2:**
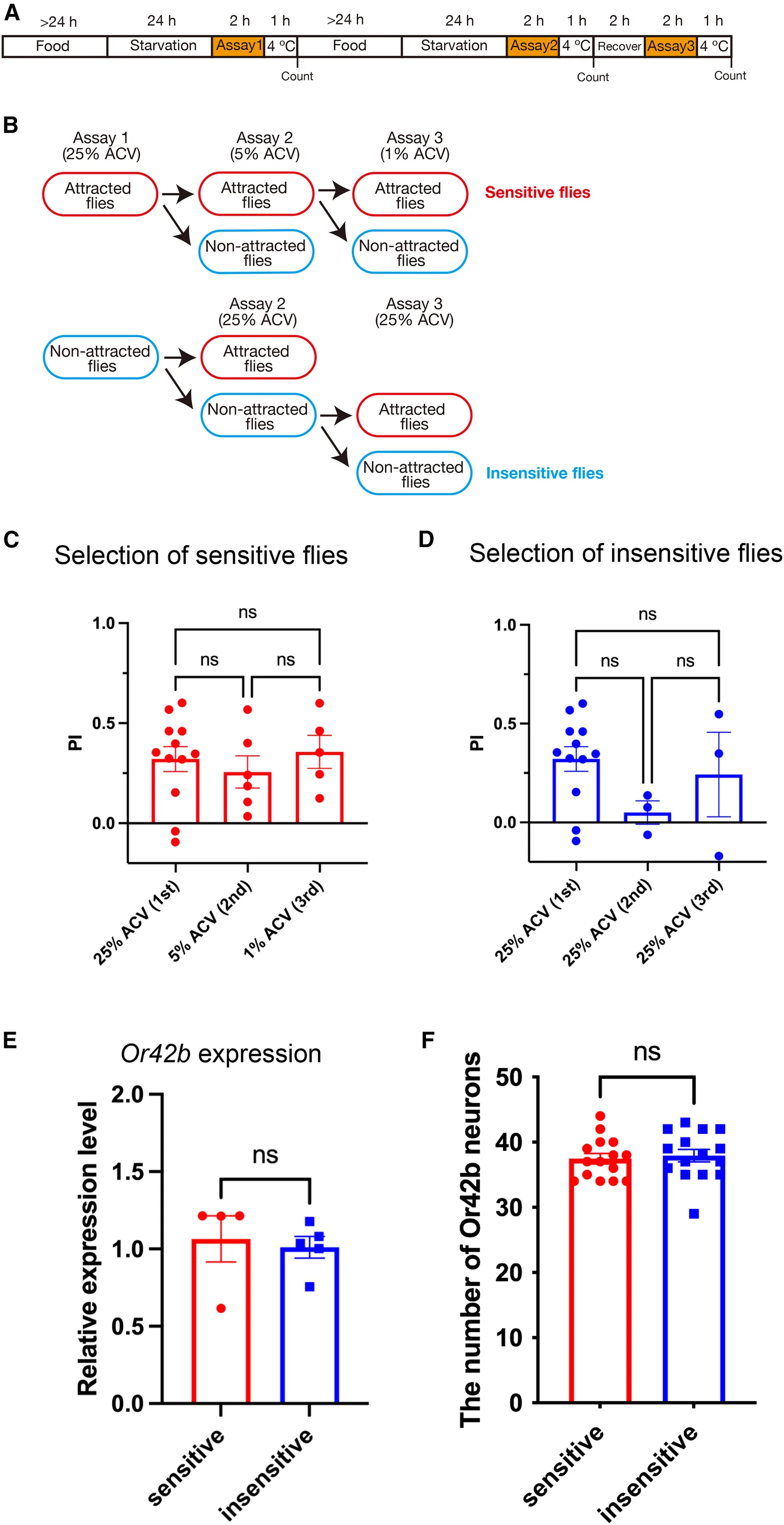
The expression level of *Or42b* and the number of Or42b neurons were not correlated with individual variations in olfactory sensitivity (A) Time course of the sequential trap assays. Flies were starved for 24 h before the first assay. After the first assay, flies were anesthetized at 4°C for 1 h and counted, and then transferred to a new vial with food for recovery for at least 24 h. Flies were then starved again for 24 h before the second assay. The second and third assays were performed on the same day, with a 2-h recovery period at 25°C without food between assays. (B) Strategy for the sequential trap assays. Sensitive flies were selected by sequentially collecting attracted flies using 25%, 5%, and 1% ACV in the first, second, and third assays, respectively, whereas insensitive flies were selected by sequentially collecting non-attracted flies using 25% ACV in all three assays. (C) PIs in the three sequential assays used to select sensitive flies. Data are represented as mean ± SEM. One-way ANOVA with Šidák’s multiple comparisons test; ns, not significant; 1st, *N* = 12; 2nd, *N* = 6; and 3rd, *N* = 5. N represents the number of independent trap assays. (D) PIs in the three sequential assays used to select insensitive flies. Data are represented as mean ± SEM. One-way ANOVA with Šidák’s multiple comparisons test; ns, not significant; 1st, *N* = 12; 2nd, *N* = 3; and 3rd, *N* = 3. N represents the number of independent trap assays. (E) The mRNA level of *Or42b* was measured by quantitative reverse transcription-polymerase chain reaction. *Gapdh2* was used as an internal control. No significant difference in the *Or42b* expression level was detected between the sensitive and insensitive flies. Data are represented as mean ± SEM. Unpaired *t* test; ns, not significant; *p* = 0.7590; sensitive, *N* = 4; and insensitive, *N* = 5. N represents the number of independent biological replicates. (F) Number of Or42b-expressing neurons in the antennae of sensitive and insensitive flies. No significant difference was detected between the two groups. Data are represented as mean ± SEM. Unpaired *t* test; ns, not significant; *p* = 0.7119; *N* = 15. N represents the number of antennae analyzed. See also Figure S1.

First, we speculated that the expression level of Or42b, the olfactory receptor for ACV, and the number of Or42b neurons may differ between sensitive and insensitive flies to ACV. We examined the expression of the *Or42b* gene and found no significant differences between the sensitive and insensitive flies (Figures 2E and S1). For normalization, we used *Gapdh2* as the internal control. We confirmed that the expression of *Gapdh2* did not differ between sensitive and insensitive flies (Figure S1A). We also counted the number of Or42b neurons using a transgenic line expressing *UAS-mCD8::PARP::Venus* under the control of *Or42b-Gal4*. No significant difference in the number of Or42b neurons was observed between sensitive and insensitive flies (Figures 2F, S1B, and S1C). Furthermore, we examined the genome sequences of *Or42b* in sensitive and insensitive flies. We examined five animals from each group, and the DNA sequences of the *Or42b* gene region were found to be identical between the two groups. This indicates that polymorphism in the *Or42b* gene is not the cause of variations in olfactory sensitivity to ACV. Therefore, these results suggest that the amounts of the olfactory receptor and olfactory receptor neurons are not related to individual variations, and there may be other factors that affect the individual differences in olfactory sensitivity toward food-related odors.

### Transcriptome analysis revealed altered lipid-related gene expression and a starvation-like transcriptional signature in sensitive flies

To examine the differences in gene expression between sensitive and insensitive flies, we performed transcriptome analysis using whole-body samples with three biological replicates for each group (Table S1). Principal component analysis (PCA) revealed clear separation between sensitive and insensitive flies, with PC1 and PC2 accounting for 51.7% and 14.3% of the total variance, respectively (Figure 3A). Analysis of gene contributions to PC1 identified the top 10 contributing genes, including *Npc2d*, a gene associated with sterol transport (Figure S2). We compared the transcriptomes of the sensitive and insensitive groups and identified 412 differentially expressed genes (DEGs), of which 92 genes were upregulated and 320 genes were downregulated in sensitive flies compared to insensitive flies (Figure 3B; Table S2). Among these DEGs, several chemosensory genes were identified, including olfactory receptor genes (*Or59c* and *Or82a*), odorant-binding protein genes (*Obp50c*, *Obp56f*, *Obp99b*, *Obp99c*, and *Obp99d*), and gustatory receptor genes (*Gr47b*, *Gr63a*, and *Gr98a*). Most of these genes were downregulated in sensitive flies, whereas *Gr63a* was upregulated (Figure 3C). Gene Ontology (GO) enrichment analysis showed that genes associated with responses to metal ions and detoxification were enriched among genes upregulated in sensitive flies (Figure 3D). In contrast, genes involved in lipid catabolism, lipid metabolism, fatty acid metabolism, and lipid transport were enriched among the downregulated genes in sensitive flies (Figures 3E–3G). These changes in lipid-related gene expression suggest that sensitive flies may differ in their metabolic or nutritional states from insensitive flies.

**Figure 3:**
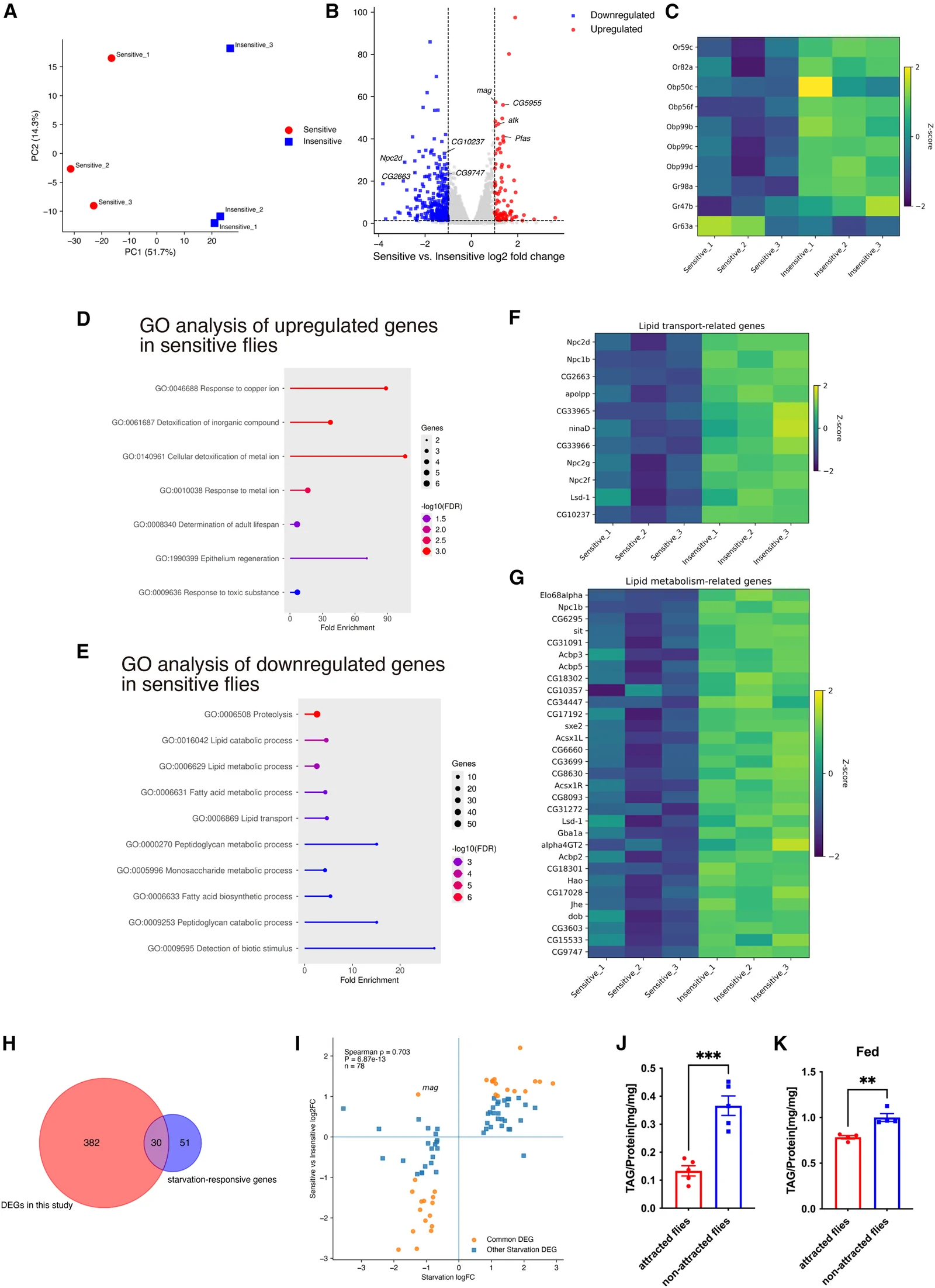
Transcriptome analysis between sensitive and insensitive flies (A) Principal component analysis (PCA) of RNA-seq data from sensitive (red circles) and insensitive (blue squares) flies. PC1 and PC2 explain 51.7% and 14.3% of the total variance, respectively. (B) Volcano plot shows differentially expressed genes (DEGs) between sensitive and insensitive flies. Genes with FDR <0.05 and |log2 fold change| ≥ 1 were considered differentially expressed. Red circles and blue squares indicate genes upregulated and downregulated in sensitive flies, respectively. The four lipid-related genes and the four genes overlapping with previously reported starvation-responsive genes^14^ with the lowest FDR values are labeled. (C) Heatmaps show the expression of DEGs associated with olfactory receptors, odorant-binding proteins, and gustatory receptors. Expression values are shown as row-wise Z-scores across the six samples. (D and E) Gene Ontology (GO) analysis of upregulated (D) and downregulated (E) genes in sensitive flies. Significantly enriched GO biological process terms are shown (FDR <0.05). The x axis indicates fold enrichment, circle size represents the number of differentially expressed genes associated with each GO term, and color represents −log10(FDR). GO terms are ordered according to FDR. (F and G) Heatmaps showing expression of DEGs associated with lipid transport (F) and lipid metabolism (G). Expression values are shown as row-wise Z-scores across the six samples. (H) Venn diagram showing the overlap between DEGs in sensitive flies and previously reported starvation-responsive genes.^14^ Thirty genes were shared between the two datasets. (I) Comparison of log fold changes for the 78 starvation-responsive genes that were also included in our RNA-seq dataset. The x axis shows the log fold change between starved and control flies reported by Moskalev et al.^14^ and the y axis shows the log2 fold change between sensitive and insensitive flies. Spearman’s rank correlation test; ρ = 0.703, *p* = 6.87 × 10^−13^, *N* = 78 genes. Orange circles indicate common DEGs, and blue squares indicate other starvation-responsive genes. (J and K) Quantification of triacylglycerol (TAG) of attracted (red) and non-attracted (blue) flies under starved (J) and fed conditions (K). Data are represented as mean ± SEM. Unpaired *t* test; ∗∗∗*p* = 0.0004, *N* = 5 (J) and ∗∗*p* = 0.0035, *N* = 4 (K). N represents the number of independent biological replicates. See also Figure S2; Tables S1, S2, and S3.

We next asked whether differences in olfactory sensitivity were associated with differences in nutritional state. We compared the DEGs identified in this study with starvation-responsive genes reported previously.^14^ Among the 81 starvation-responsive genes, 30 were also differentially expressed between sensitive and insensitive flies (Figure 3H; Table S3). Of these 30 overlapping genes, 29 showed concordant directions of expression change between sensitive flies and under starvation. To further assess the relationship between the two datasets, we examined 78 starvation-responsive genes that were also included in our RNA-seq dataset. Their starvation-associated log fold changes showed a strong positive correlation with the log2 fold changes between sensitive and insensitive flies (Spearman’s *ρ* = 0.703, *p* = 6.87 × 10^−13^; Figure 3I). These results indicate that sensitive flies exhibit a transcriptional signature that partially resembles the response to starvation.

Since genes involved in lipid metabolism were downregulated in sensitive flies, we measured the amount of triacylglycerol (TAG), an important energy source.^15^ For TAG measurements, attracted and non-attracted flies were collected using a single olfactory assay. Even after a single assay, TAG levels were significantly lower in attracted flies than in non-attracted flies (Figure 3J). Furthermore, even after feeding for 45 h after the assay, TAG levels remained significantly lower in attracted flies (Figure 3K). These results suggest that differences in olfactory sensitivity are associated with differences in energy storage and nutritional state.

### sNPF/sNPF-R signaling is associated with individual differences in ACV sensitivity

Starvation is one of the important factors regulating food-seeking behavior.^5^^,^^6^ A previous study reported that starvation-dependent food-seeking behavior is mediated by sNPF signaling and that starvation increases the expression of sNPF-R, a short neuropeptide F receptor in Or42b neurons, in response to insulin signaling.^5^ We hypothesized that sensitive flies are more starved than insensitive flies and that a higher starvation level induces higher sensitivity to food odors, which is modulated by sNPF-R. To test this hypothesis, we examined the expression levels of *sNPF* and *sNPF-R* in sensitive and insensitive flies. The expression level of *sNPF* in the head was not significantly different between the two types of flies (Figure 4A), which is consistent with the previous study showing that starvation did not change the expression of *sNPF*.^5^ In contrast, the *sNPF-R* level in sensitive flies was significantly higher than that in insensitive flies (Figure 4B). These results suggest that there are individual differences in starvation levels, and the internal physiological state is likely to correlate with olfactory sensitivity to food odors.

**Figure 4:**
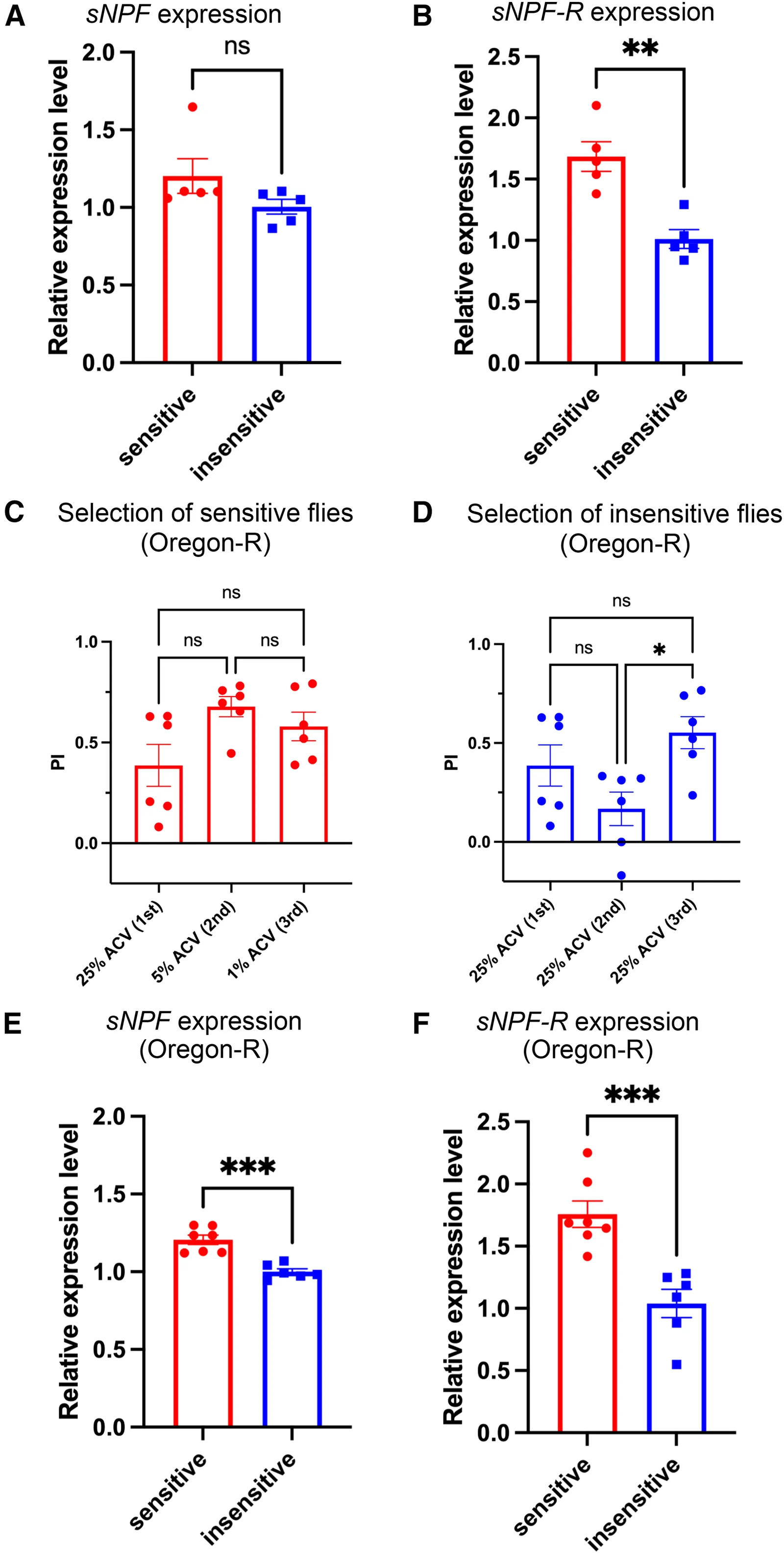
*sNPF-R* was upregulated in sensitive flies (A and B) Relative expression levels of *sNPF* (A) and *sNPF-R* (B). *Gapdh2* was used as an internal control. There was no significant difference between the sensitive and insensitive flies with respect to the *sNPF* expression level but the expression level of *sNPF-R* in sensitive flies was higher than that in insensitive flies. Data are represented as mean ± SEM. Unpaired *t* test; ns, not significant, *p* = 0.1605; *N* = 5 (A), ∗∗*p* = 0.0025; *N* = 5 (B). N represents the number of independent biological replicates. (C and D) PIs in the three sequential assays used to select sensitive (C) and insensitive (D) flies of the Oregon-R strain. Data are represented as mean ± SEM. One-way ANOVA with Šidák’s multiple comparisons test; ns, not significant; ∗*p* = 0.0263; *N* = 6. N represents the number of independent trap assays. (E and F) Relative expression levels of *sNPF* (E) and *sNPF-R* (F) in the Oregon-R strain. *Act5c* was used as an internal control. The expression levels of *sNPF* and *sNPF-R* were higher in sensitive flies than in insensitive flies. Data are represented as mean ± SEM. Unpaired *t* test; ∗∗∗*p* = 0.0002 (E); ∗∗∗*p* = 0.0008 (F); sensitive, *N* = 7; insensitive, *N* = 6. N represents the number of independent biological replicates.

To examine whether the differential expression of *sNPF-R* was also observed in another genetic background, we performed the same selection assays using another wild-type strain, Oregon-R flies. Using the same selection procedure, we were also able to separate Oregon-R flies into sensitive and insensitive groups (Figures 4C and 4D). We then compared *sNPF* and *sNPF-R* expression between the two groups. The expression levels of both *sNPF* and *sNPF-R* expressions were significantly higher in sensitive Oregon-R flies than in insensitive flies (Figures 4E and 4F). These results suggest that the association of the sNPF/sNPF-R signaling pathway with individual differences in ACV preference is conserved across different genetic backgrounds, although the regulation of individual pathway components may vary among strains.

### Starvation contributes to individual differences in olfactory sensitivity to ACV

If sensitive flies are more starved than insensitive flies, the former would be more vulnerable to starvation. To address this possibility, we performed a starvation assay after the first trap assay (Figure 5A). The attracted flies died significantly earlier than the non-attracted flies (Figure 5B), thus supporting our hypothesis that sensitive flies are more vulnerable to starvation.

**Figure 5:**
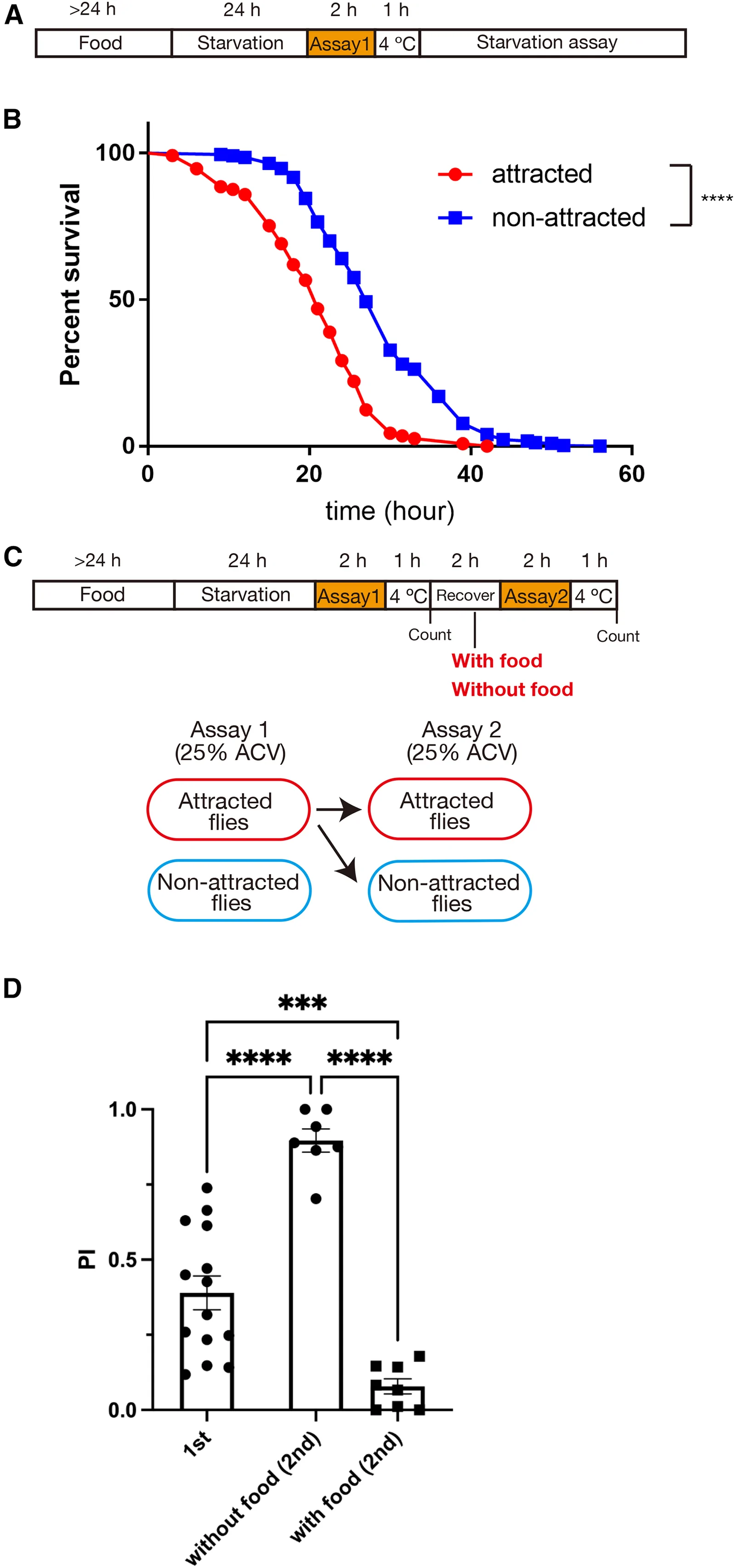
Starvation level affects the olfactory sensitivity to apple cider vinegar (A) Time course of the trap and starvation assays. The starvation assay was performed using attracted and non-attracted flies selected by the trap assay. (B) Representative results of the starvation assay using attracted flies (red, *N* = 113) and non-attracted flies (blue, *N* = 400). Attracted flies were more sensitive to starvation than non-attracted flies. Gehan-Breslow-Wilcoxon test; ∗∗∗∗*p* < 0.0001. N represents the number of individual flies. The experiment was repeated three times. (C) Time course of the sequential trap assays. Attracted flies were recovered after the first assay with or without food and used for the second assay. (D) The average PI of flies without or with food during recovery is shown. Data are represented as mean ± SEM. One-way ANOVA with Šidák’s multiple comparisons test; ∗∗∗*p* = 0.0005; ∗∗∗∗*p* < 0.0001; 1st, *N* = 14; without food, *N* = 7; with food, *N* = 8. N represents the number of independent trap assays.

To confirm whether the individual starvation level is involved in the olfactory differences between the sensitive and insensitive flies, we collected flies attracted to 25% ACV in the first trap assay and maintained the flies with or without food during the recovery time before the second trap assay (Figure 5C). Without food during the recovery time, the PIs increased as compared to the PIs of the first assay (Figure 5D). On the other hand, the flies fed during the 2-h recovery time showed significantly decreased PIs in the second assay (Figure 5D). Thus, sensitive flies appear to be in a more starved physiological state and to express higher levels of *sNPF-R*, suggesting that differences in internal state underlie individual variation in olfactory sensitivity.

## Discussion

The mechanism underlying individual variation in behavior is a fundamental question in neuroscience but remains incompletely understood. Here, we showed that some flies were relatively sensitive, while others were relatively insensitive to food-related odors, even though they shared the same genetic background and were maintained under the same standard laboratory conditions (Figure 1). The differences could not be explained by the expression level or DNA sequence of *Or42b* or by the number of Or42b-positive olfactory receptor neurons (Figure 2). Transcriptome analysis between sensitive and insensitive flies revealed differential expression of several chemosensory genes and downregulation of genes related to lipid metabolism in sensitive flies (Figure 3). TAG levels also remained lower in attracted flies than in non-attracted flies even after feeding (Figure 3). A previous study showed that starvation increases *sNPF-R* expression and that *sNPF-R* promotes the response of Or42b neurons and food-searching behavior.^5^ Consistent with this, we found higher *sNPF-R* expression in sensitive flies (Figure 4). Moreover, sensitive flies were more vulnerable to starvation conditions, and feeding attracted flies during the recovery period between two trap assays markedly reduced ACV preference in the subsequent assay, supporting a link between individual differences in olfactory responsiveness and nutritional state (Figure 5). Although the differential expression of chemosensory genes suggests that additional sensory mechanisms may also contribute to the observed variation, our results suggest that differences in nutritional state are associated with individual variation in *sNPF-R* expression, which may contribute to differences in olfactory sensitivity to food-related odors.

### Chemosensory genes differentially expressed between sensitive and insensitive flies

RNA-seq analysis identified several differentially expressed chemosensory genes, including olfactory receptor (*Or59c* and *Or82a*), odorant-binding protein (*Obp50c*, *Obp56f*, *Obp99b*, *Obp99c*, and *Obp99d*), and gustatory receptor genes (*Gr47b*, *Gr63a*, and *Gr98a*) (Figure 3C). Although none of these genes has been directly implicated in ACV preference, their differential expression raises the possibility that changes in peripheral chemosensory function contribute to the individual variation in ACV sensitivity observed in this study.

Odorant-binding proteins (OBPs) are small soluble proteins that bind odorants and can modulate chemosensory responses.^16^ Five *Obp* genes were differentially expressed in our RNA-seq analysis, including *Obp99b*, *Obp99c*, and *Obp99d*, which were downregulated in sensitive flies. Natural polymorphisms in the *Obp99* genes have previously been associated with variation in olfactory responses to benzaldehyde and acetophenone.^17^^,^^18^ Thus, altered expression of these OBPs, together with differentially expressed *Or* genes, may contribute to differences in odorant sensitivity between sensitive and insensitive flies. Further functional analyses will be required to determine whether these genes contribute to responses to ACV.

An alternative, but not mutually exclusive, possibility is that some of these transcriptional differences reflect physiological differences outside the olfactory system. Because our RNA-seq analysis was performed using whole-body samples, changes in expression do not necessarily reflect altered expression in olfactory tissues. Several genes identified in our analysis are also expressed in non-olfactory tissues. For example, *Or59c*, *Obp50c*, and *Obp56f* are expressed in male reproductive tissues, including testis and accessory gland.^19^^,^^20^^,^^21^ The expression of OBP genes is also sensitive to nutritional state. Notably, *Obp99b*, which was downregulated in sensitive flies in our study, is downregulated in males under starvation.^22^ In addition, genetic analysis of the *Obp50a-d* cluster has revealed sex-and mating-status-dependent effects on starvation resistance.^20^ Thus, the reduced expression of these genes in sensitive flies may reflect differences in nutritional or physiological state rather than changes restricted to the olfactory system. This possibility is consistent with our hypothesis that sensitive flies are in a more nutritionally deprived state.

### Differences in lipid metabolism and sNPF-R signaling are associated with individual variation in olfactory sensitivity

GO enrichment analysis of DEGs downregulated in sensitive flies identified significant enrichment of terms related to lipid metabolism and lipid transport (Figure 3E). Consistent with this, TAG levels were lower in attracted flies than in non-attracted flies even after feeding (Figures 3J and 3K). Attracted flies also showed reduced resistance to starvation (Figure 5B). Together, these metabolic and behavioral phenotypes suggest that sensitive flies may have lower energy reserves and may be in a physiological state resembling greater nutritional deprivation. Supporting this interpretation, the expression changes of starvation-responsive genes showed a strong positive correlation with those observed between sensitive and insensitive flies (Figure 3I), suggesting that the transcriptional state of sensitive flies partially resembles the response to starvation (Figures 3H and 3I).

Interestingly, *mag* was the only overlapping gene that showed the opposite pattern, being upregulated in sensitive flies but downregulated in the starvation dataset (Figure 3I; Table S3). *mag* encodes a homolog of mammalian lysosomal acid lipase (LipA) and is expressed in the anterior intestine, where the protein is secreted into the intestinal lumen and promotes the digestion of dietary triacylglycerol.^23^ Loss of *mag* impairs dietary lipid utilization and reduces whole-body TAG stores, whereas restoring *mag* expression can recover reduced TAG levels under conditions in which *mag* expression is low.^23^^,^^24^ Therefore, the increased expression of *mag* in sensitive flies is unlikely to directly explain their lower TAG levels. Instead, its upregulation may represent a compensatory response to reduced lipid reserves, potentially enhancing the utilization of dietary lipids.

sNPF/sNPF-R signaling provides a possible link between nutritional state and olfactory sensitivity. Starvation increases transcription of *sNPF-R* and enhances presynaptic activity in Or42b olfactory receptor neurons through sNPF signaling, thereby promoting odor-driven food-search behavior.^5^ Expression of sNPF and sNPF-R in Or42b neurons is required for this starvation-dependent behavioral modulation, and insulin signaling suppresses *sNPF-R* expression, directly linking metabolic state to olfactory sensitivity.^5^ In this study, *sNPF-R* expression was higher in sensitive flies in both Canton-S and Oregon-R (Figure 4), indicating that this association is not restricted to a single genetic background and is consistent with the starvation-dependent upregulation of *sNPF-R* reported previously.^5^ In contrast, *sNPF* expression showed strain-dependent differences: it was increased in sensitive Oregon-R flies but was not significantly altered in Canton-S (Figure 4). Root et al. also detected no starvation-induced change in *sNPF* transcript levels.^5^ Thus, increased *sNPF-R* expression appears to be the more consistent molecular feature associated with enhanced olfactory sensitivity, whereas regulation of *sNPF* expression may depend on genetic background or physiological context.

Notably, *sNPF-R* was not identified as a DEG in our RNA-seq analysis. One possible explanation is the difference in tissue composition between the two analyses: RNA-seq was performed using whole bodies, whereas qPCR analysis of *sNPF-R* was performed using heads. A modest change in *sNPF-R* expression in a restricted population of neurons may therefore have been diluted in whole-body transcriptome data. Further analysis will be required to determine whether the neural responses of Or42b neurons and downstream neurons differ systematically between sensitive and insensitive flies.

In vertebrates, neuropeptide Y (NPY), distantly related to *Drosophila sNPF*, plays an important role in the regulation of food intake and energy homeostasis.^25^ Interestingly, NPY modulates olfactory responses to food-related odors in hungry axolotls and rats.^26^^,^^27^ Treatment with NPY increases the olfactory response, and the expression level of the NPY receptor is upregulated in fasted rats.^27^ In addition to the acute modulation of the olfactory response, NPY promotes the proliferation and differentiation of olfactory receptor neurons.^28^^,^^29^ Although we did not observe a difference in the number of Or42b neurons between sensitive and insensitive flies, the number of olfactory receptor neurons modulated by NPY signaling could affect individual differences in olfactory sensitivity in vertebrates. Moreover, NPY expression levels vary among individuals because of genetic variations in humans.^30^ The variations in olfactory sensitivity modulated by sNPF and NPY signaling may be conserved among vertebrates and invertebrates.

### Limitations of the study

Although sensitive and insensitive flies shared the same genetic background and were maintained under the same standard laboratory conditions, the factors generating differences in their physiological state remain unclear. Subtle differences in developmental conditions may contribute to such variation. For example, larval density affects adult body size and several physiological traits, including fat reserves and starvation resistance, in *Drosophila*.^31^ In our study, the number of eggs or larvae developing in each culture vial was not strictly controlled. Thus, differences in developmental history may contribute to the physiological variation observed among adult flies.

Importantly, sensitive and insensitive flies were classified through sequential trap assays performed over more than one day, with recovery and feeding periods between assays. Therefore, the observed behavioral differences are unlikely to reflect only transient changes in physiological state. Instead, they suggest that relatively persistent differences in metabolic or physiological state may underlie the behavioral variation. Further studies will be required to determine how such differences are established and maintained.

The relationship observed in this study suggests that individual differences in physiological state may contribute to individual variation in olfactory sensitivity, although whether this relationship extends to odors other than ACV remains unknown. Olfactory responses in *Drosophila* are influenced by the physiological or behavioral state of the animal. For example, behavioral responses to the male-specific pheromone *cis*-vaccenyl acetate (cVA) depend on social context and previous courtship experience.^32^ Thus, individual variation in the physiological state relevant to each odor may underlie individual differences in olfactory responses to distinct classes of odors. An important next step will be to identify the molecular and neural mechanisms by which such physiological differences are translated into individual variation in olfactory sensitivity. Determining how distinct internal-state signals modulate specific sensory pathways may reveal whether different forms of behavioral individuality arise through common or stimulus-specific mechanisms.

## Resource availability

### Lead contact

Requests for further information and resources should be directed to and will be fulfilled by the lead contact, Misako Okumura (misako.okumura.c2@tohoku.ac.jp).

### Materials availability

This study did not generate new unique reagents.

### Data and code availability

The RNA-seq data generated in this article have been deposited in the DDBJ BioProject database under accession number BioProject: PRJDB17319 (https://ddbj.nig.ac.jp/search/entry/bioproject/PRJDB17319). This paper does not report original code. Any additional information required to reanalyze the data reported in this paper is available from the lead contact upon request.

## Acknowledgments

We would like to thank Bloomington Drosophila Stock Center (NIHP40OD018537) for providing the fly strains, Dr. Yuuki Ishita, Mr. Wei Wang, and Mr. Hirokuni Hiraga (10.13039/501100003790Hiroshima University) for their technical help, all the members of Chihara laboratory for their kind support, and Editage (www.editage.jp) for English language editing. This work was supported by 10.13039/501100001691JSPS 10.13039/501100001691KAKENHI Grant Numbers 18H05369 and 20K20378 to T.C. Furthermore, the 10.13039/501100022598Toray Science Foundation, The Frontier Development Program for Genome Editing, and Program for Advancing Strategic International Networks to Accelerate the Circulation of Talented Researchers JPMXS05S2900002 provided support to T.C.

## Author contributions

Conceptualization, M.O., A.I., Y.S., M.N.D., and T.C.; methodology, M.O., Y.S., M.N.D.; investigation, M.O., A.I., Y.U., Y.S., and M.N.D.; writing – original draft, M.O.; writing – review and editing, all other authors; funding acquisition, T.C.; supervision, T.C.

## Declaration of interests

The authors declare no competing interests.

## Declaration of generative AI and AI-assisted technologies in the manuscript preparation process

During the preparation of this work, the authors used ChatGPT in order to assist with writing Python code and translating and editing English text. After using this tool or service, the authors reviewed and edited the content as needed and take full responsibility for the content of the publication.

## STAR★Methods

### Key resources table

**Table:** 

REAGENT or RESOURCE	SOURCE	IDENTIFIER
**Antibodies**		

Rat anti-mouse CD8	Thermo Fisher Scientific	Cat#MCD0800; RRID:AB_10392843
Goat anti-Rat IgG (H + L) Cross-Adsorbed Secondary Antibody, Alexa Fluor 488	Thermo Fisher Scientific	Cat#A-11006; RRID:AB_2534074

**Critical commercial assays**		

LabAssay^TM^ Triglyceride	Fujifilm Wako Pure Chemicals Corporation	Cat#632-50991
BCA Protein Assay Kit	TaKaRa Bio	T9300A

**Deposited data**		

RNA-seq raw data	This paper	DDBJ: PRJDB17319; https://ddbj.nig.ac.jp/search/entry/bioproject/PRJDB17319
Drosophila melanogaster reference genome (BDGP6)	Ensembl	Ensembl, BDGP6

**Experimental models: Organisms/strains**		

Canton-S	Bloomington Drosophila Stock Center	RRID:BDSC_64349
Oregon-R-SNPiso3	Bloomington Drosophila Stock Center	RRID:BDSC_6363
*Or42b-Gal4*	Bloomington Drosophila Stock Center	RRID:BDSC_9971
*UAS-mCD8::PARP::Venus*	Williams et al.^33^	N/A

**Oligonucleotides**		

Primers for qRT-PCR, see Table S4	This paper	N/A
Primers for *Or42b* sequencing, see Table S4	This paper	N/A

**Software and algorithms**		

Fiji	Schindelin et al.^34^	https://fiji.sc/
HISAT2	Kim et al.^35^	https://daehwankimlab.github.io/hisat2/
StringTie	Pertea et al.^36^	https://ccb.jhu.edu/software/stringtie/index.shtml
iDEP	Ge et al.^37^	https://bioinformatics.sdstate.edu/idep/
DESeq2	Love et al.^38^	https://bioconductor.org/packages/release/bioc/html/DESeq2.html
Python	Python Software Foundation	https://www.python.org/
GraphPad Prism	GraphPad Software	https://www.graphpad.com/

### Experimental model and study participant details

#### Fly strains and culture conditions

*Drosophila melanogaster* used in this study were raised under standard laboratory conditions at 25°C. The following strains were used: Canton-S (RRID:BDSC_64349), Oregon-R-SNPiso3 (RRID:BDSC_6363) and *Or42b-Gal4* obtained from the Bloomington Drosophila Stock Center and *UAS-mCD8::PARP::Venus*.^33^ Adult male flies aged 1-3 days after eclosion were used at the beginning of the experimental procedures. Because only male flies were used, potential sex-dependent differences could not be assessed. The experiments were approved by the Biosafety Committee for Living Modified Organisms, Hiroshima University, and were performed in accordance with the Japanese law, Act on the Conservation and Sustainable Use of Biological Diversity through Regulations on the Use of Living Modified Organisms.

### Method details

#### Olfactory trap assay

1-3-day-old adult male flies were divided into 25 individuals per vial with food for at least 24 h and starved for 24 h before the first assay at 25°C and 60% humidity under 12:12-h light:dark conditions. Olfactory trap assays were performed as previously described, with some modifications^39^ (Figure 1A). A 1000-mL glass beaker was covered with gauze, and two glass bottles were placed inside the beaker. Two pieces of paper (Kimwipes, Crecia) soaked with 500 μL of diluted ACV (Mizkan Co., Ltd., Japan; 5% acidity) or distilled water were placed in two respective glass bottles. ACV concentrations indicate dilutions of the original commercial ACV solution. The glass bottles were covered with a foam plug into which a truncated pipette tip was inserted (Mixell, MIX-Y110B, 200-μL tip; cut approximately 10 mm from the tip, with an opening diameter of approximately 2 mm), allowing flies to enter the glass bottle. For Figure 1F, gauze was placed between the truncated pipette tip and the glass bottle, allowing flies to detect the odor and enter through the pipette tip while preventing them from reaching the bottom of the bottle and ingesting ACV or water (Figure 1E). The beakers with approximately 100 starved males were kept for 2 h in the dark at 25°C and 60% humidity. The number of dead flies in the beaker was counted after 2 h, and the beakers were kept at 4°C for 1 h to anesthetize the flies. The number of flies in the ACV trap, control trap, and field were counted to calculate the PI as follows:

PI = (No. of flies in the ACV trap – No. of flies in the control trap)/(Total No. of flies − No. of dead flies).

Flies in the ACV trap were collected as attracted flies, and those in the control trap and field were collected as non-attracted flies.

For sequential assays, flies collected from the previous assay were re-assayed. In Figure 1, flies were tested three times using 25% ACV or water. The first and second assays were performed on the same day with a 2-h recovery at 25°C without food between assays. The third assay was performed after flies had been transferred to normal food vials for at least 24 h and then starved again for 24 h before the assay. For the second and third assays in these experiments, flies collected from the previous assays were combined to obtain sufficient numbers of flies, and assays containing more than 11 individuals per beaker were included in the analysis.

In Figures 2 and 4, sensitive flies were collected by sequentially selecting attracted flies with 25%, 5%, and 1% ACV, whereas insensitive flies were collected by sequentially selecting non-attracted flies in three assays using 25% ACV. In these experiments, flies were transferred to normal food vials after the first assay, allowed to recover for at least 24 h, and then starved again for 24 h before the second assay. The second and third assays were performed on the same day with a 2-h recovery period at 25°C without food between assays.

For the RNA-seq experiments shown in Figure 3, all three sequential assays were performed on different days. After each assay, flies were allowed to recover on normal food for at least 24 h and were then starved again for 24 h before the subsequent assay. The other conditions were the same as those used for the sequential assays in Figures 2 and 4.

For the TAG and survival assays shown in Figures 3J, 3K, 5A, and 5B, attracted and non-attracted flies were collected after a single assay using 25% ACV. For Figure 3K, flies were transferred to normal food vials and fed for 45 h before TAG measurement. For Figures 5C and 5D, the first and second assays were performed on the same day using 25% ACV, with a 2-h recovery period either with or without food.

#### Immunostaining and imaging of antennae

Immunostaining of fly antennae was performed as previously described, with some modifications.^40^ Briefly, the fly antennae were cut off in 0.3% PBST (PBS with 0.3% Triton X-100) using forceps. The dissected antennae were transferred to a fixation solution (4% paraformaldehyde in 0.1% PBST) and incubated for 30 min at room temperature. The antennae were washed twice in 100 μL of 0.3% PBST, and then washed for 20 min three times in 100 μL of 0.3% PBST. The antennae were blocked with 100 μL of 5% normal goat serum (NGS) in 0.1% PBST for 60 min at room temperature. After removing the blocking solution, the antennae were incubated with 100 μL of a primary antibody (Anti-mCD8, rat, Invitrogen, MCD0800, 1:100) in 0.1% PBST containing 5% NGS for 48 h at 4°C. The antennae were washed as previously mentioned, and incubated with 100 μL of a secondary antibody (Goat Anti-Rat IgG Alexa Fluor 488, ThermoFisher Scientific, A-11006, 1:250) in 0.1% PBST containing 5% NGS for 48 h at 4°C. After washing the antennae, 50 μL of SlowFade Diamond Antifade Mountant (Life Technologies, S36972) was added. The antennae were then mounted on a slide. The samples were imaged using a laser scanning microscope (ZEISS LSM 5 PASCAL), and the number of cell bodies was counted manually using the ImageJ software.^34^

#### Quantitative reverse transcription polymerase chain reaction (qRT-PCR)

Total RNA was isolated from 10 fly heads using TRIzol reagent (Invitrogen, 15596018), according to the manufacturer’s protocol. Genomic DNA was eliminated, and cDNA was synthesized using the PrimeScript^TM^ RT reagent kit with gDNA Eraser (TaKaRa Bio, RR047A). Quantitative reverse transcription polymerase chain reaction (qRT-PCR) was performed using CFX Connect (BIORAD) and Luna Universal qPCR Master Mix (New England BioLabs, M3003). Three technical replicates were performed for each biological replicate. Relative expression levels were calculated using *Gapdh2* or *Act5c* as an internal control. The primers are listed in Table S4.

#### Or42b sequencing

The genomic region including Or42b was amplified by PCR using the primers listed in Table S4. The amplified region was read using the primers listed in Table S4.

#### RNA-sequencing

To perform transcriptome analysis, total RNA was extracted from sensitive or insensitive flies using TRIzol Reagent (Invitrogen, 15596018). RNA was extracted from 10 whole bodies per replicate, and three replicates were prepared for each condition. RNA quality check, sequencing, and trimming were performed by BGI Genomics (Shenzhen, China). Libraries were prepared using oligo(dT)-based mRNA enrichment and sequenced as 100-bp paired-end reads (PE100) on a DNBSEQ platform. Trimmed reads were mapped to *Drosophila* genome reference (Drosophila_melanogaster_Ensembl_BDGP6) using HISAT2 (version 2.2.1).^35^ Sequencing and alignment summary statistics are provided in Table S1.

Read counts were obtained using StringTie (version 3.0.3)^36^ with reference-guided quantification restricted to annotated transcripts (-G, -e). Gene-level count matrices were generated from StringTie output using the prepDE.py3 script provided with StringTie, with a read length of 100 bp (-l 100). Read count data were analyzed using iDEP version 2.4.4.^37^ During preprocessing, genes with at least 0.5 counts per million (CPM) in at least three libraries were retained for downstream analysis. Principal component analysis (PCA) was performed using iDEP. Gene contributions to PC1 were calculated in iDEP, and the top 10 contributing genes were identified. Differentially expressed genes (DEGs) were identified using DESeq2^38^ with FDR <0.05 and an absolute log2 fold change ≥1.

Gene Ontology (GO) enrichment analysis was performed separately for genes upregulated and downregulated in sensitive flies using iDEP. GO biological process terms with an FDR <0.05 were considered significantly enriched.

To compare transcriptional changes in sensitive flies with those induced by starvation, starvation-responsive genes reported previously^14^ were obtained from the supplementary dataset of that study. For genes represented by multiple transcripts, transcript-level log fold changes were averaged after confirming that all starvation-responsive transcripts listed for the same gene showed concordant directions of expression change. This resulted in 81 starvation-responsive genes at the gene level. For the 78 starvation-responsive genes that were also included in our expression-filtered RNA-seq dataset, the relationship between starvation-associated log fold changes and the log2 fold changes between sensitive and insensitive flies was assessed using Spearman’s rank correlation in Python.

#### TAG measurement

After conducting the olfactory trap assay as mentioned above, 10-20 flies, both attracted and non-attracted, were collected into 1.5-mL tubes. These samples were immediately frozen in liquid nitrogen and subsequently stored at −80°C. Next, 125 μL of 5% PBST was added to each tube. The samples were incubated at 70°C for 5 min, followed by centrifugation at 20,000 × G for 3 min at 4°C, and the supernatant was collected after centrifugation.

The TAG content was quantified using a Lab Assay TM Triglyceride kit (Fujifilm Wako Pure Chemicals Corporation, 632-50991). TAG levels were normalized to the total protein content, which was determined using a BCA Protein Assay Kit (TaKaRa Bio, T9300A). The absorbance at 600 nm was measured using a plate reader (Molecular Devices, SpectraMax iD3).

#### Starvation assay

To perform a starvation assay, we selected attracted and non-attracted flies using a single olfactory trap assay. After the olfactory trap assay, the flies were separated into groups of 20 animals per vial without food. A piece of paper with 500 μL distilled water was placed at the bottom of the vials. The flies were incubated at 25°C with 60% humidity under 12:12-h light:dark conditions. The numbers of dead and alive flies were counted every 1-6 h. The same experiments were performed three times.

### Quantification and statistical analysis

Data processing and statistical analyses were performed using GraphPad Prism11 software (GraphPad Software), including unpaired t-tests, one-way ANOVA followed by Šidák’s multiple comparisons test, and the Gehan-Breslow-Wilcoxon test. Details of RNA-seq differential expression and Gene Ontology enrichment analyses are described in the STAR Methods section. All data are shown as mean ± standard error of the mean (SEM), unless otherwise indicated. The statistical tests used, exact sample sizes (N), definitions of N, and statistical significance for each experiment are provided in the corresponding figure legends. Statistical significance was set at *p* < 0.05. Asterisks indicate statistical significance as follows: ∗*p* < 0.05, ∗∗*p* < 0.01, ∗∗∗*p* < 0.001, and ∗∗∗∗*p* < 0.0001. The sample size was not predetermined using statistical methods. The investigator was blinded to the experimental group when counting Or42b neurons.

## Supplemental information

Document S1. Figures S1 and S2

Table S1. RNA-seq mapping summary and gene-level read counts used for RNA-seq analysis, related to Figure 3 Summary statistics for sequencing and read alignment are provided for each RNA-seq sample, including the number of clean read pairs, primary mapped reads, primary mapping rate, uniquely mapped primary reads, and unique mapping rate. Gene-level read counts were generated from StringTie output using the prepDE.py3 script. The table includes FlyBase gene IDs, gene symbols, and read counts for three sensitive and three insensitive biological replicates

Table S2. Differential expression and enrichment analysis of sensitive and insensitive flies, related to Figure 3 The table includes DESeq2 results for all analyzed genes, the 412 differentially expressed genes (DEGs), chemosensory and lipid-related DEGs used for the heatmaps in Figures 3C, 3F, and 3G, and the results of Gene Ontology biological process enrichment analysis

Table S3. Comparison with starvation-responsive genes, related to Figure 3 The table includes starvation-responsive genes reported by Moskalev et al.^14^ and overlapping DEGs identified between sensitive and insensitive flies

Table S4. A list of primers used in this study, related to Figures 2 and 4
