# Supplementary material for: Individual variation in food odor sensitivity is related to neuropeptide signaling and starvation in Drosophila
Source: iScience. 2026 Nov 4;29(11):118105. doi: 10.1016/j.isci.2026.118105 (PMC13753345; doi:10.1016/j.isci.2026.118105)
Supplement: Supplementary file 1 [file mmc1.pdf]

iScience, Volume *29*

Supplemental information

Individual variation in food odor sensitivity is related to neuropeptide signaling and starvation in *Drosophila*

Misako Okumura, Yoshiaki Shibukawa, Yumiko Ukita, Akira Ito, Mami Nakamizo-Dojo, and Takahiro Chihara

Document S1

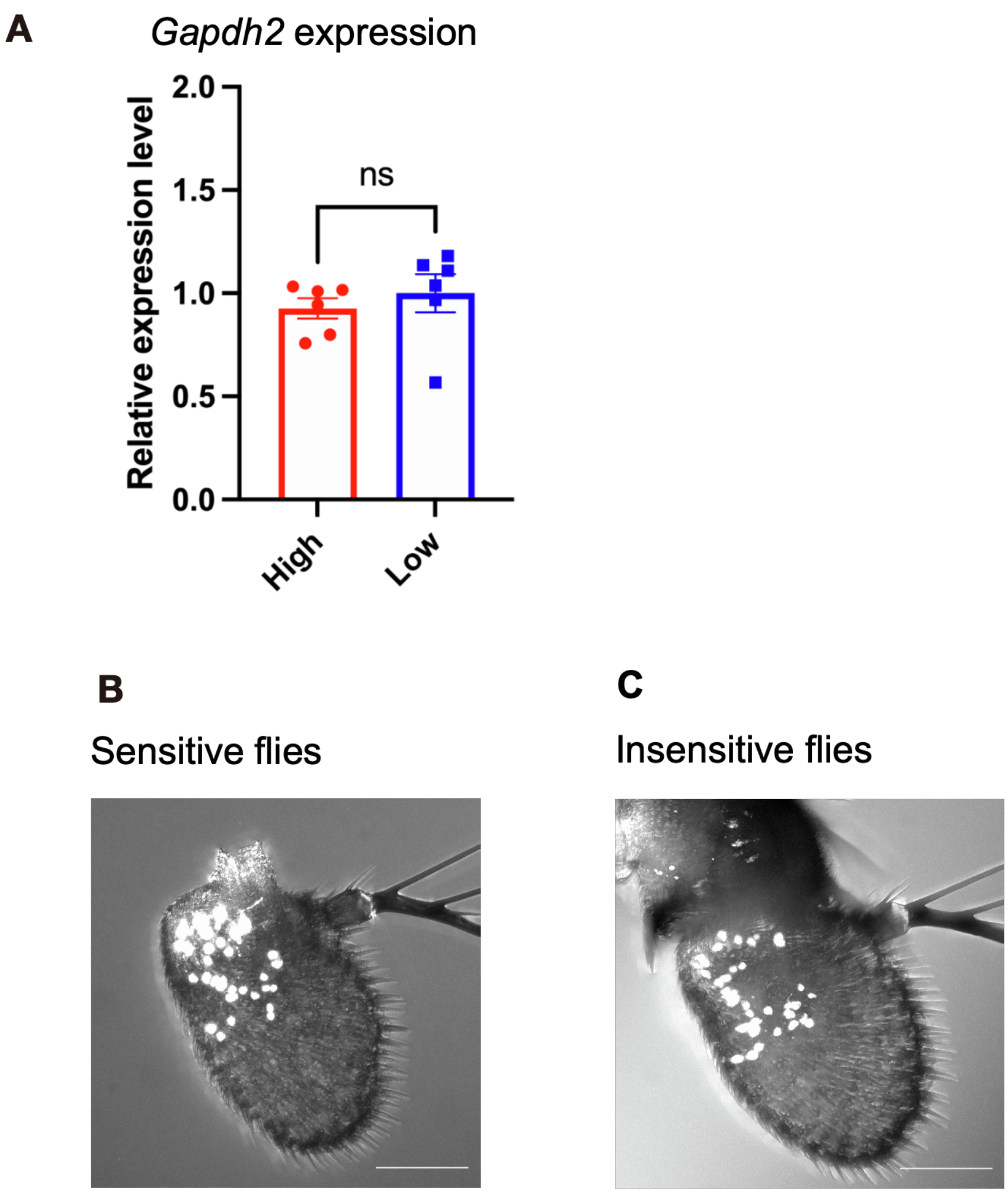

Figure S1. *Gapdh2* expression and representative images of Or42b-expressing neuron in sensitive and insensitive flies. Related to Figure 2.

(A) Relative expression levels of *Gapdh2* in sensitive and insensitive flies. *Act5c* was used as an internal control. No significant difference was detected between sensitive and insensitive flies. Data are represented as mean +/- SEM. Unpaired t-test; ns, p = 0.4950. N = 6 independent biological samples.

(B, C) Representative projection images of Or42b-expressing neurons in sensitive (B) and insensitive (C) flies. Scale bars are 50 μm.

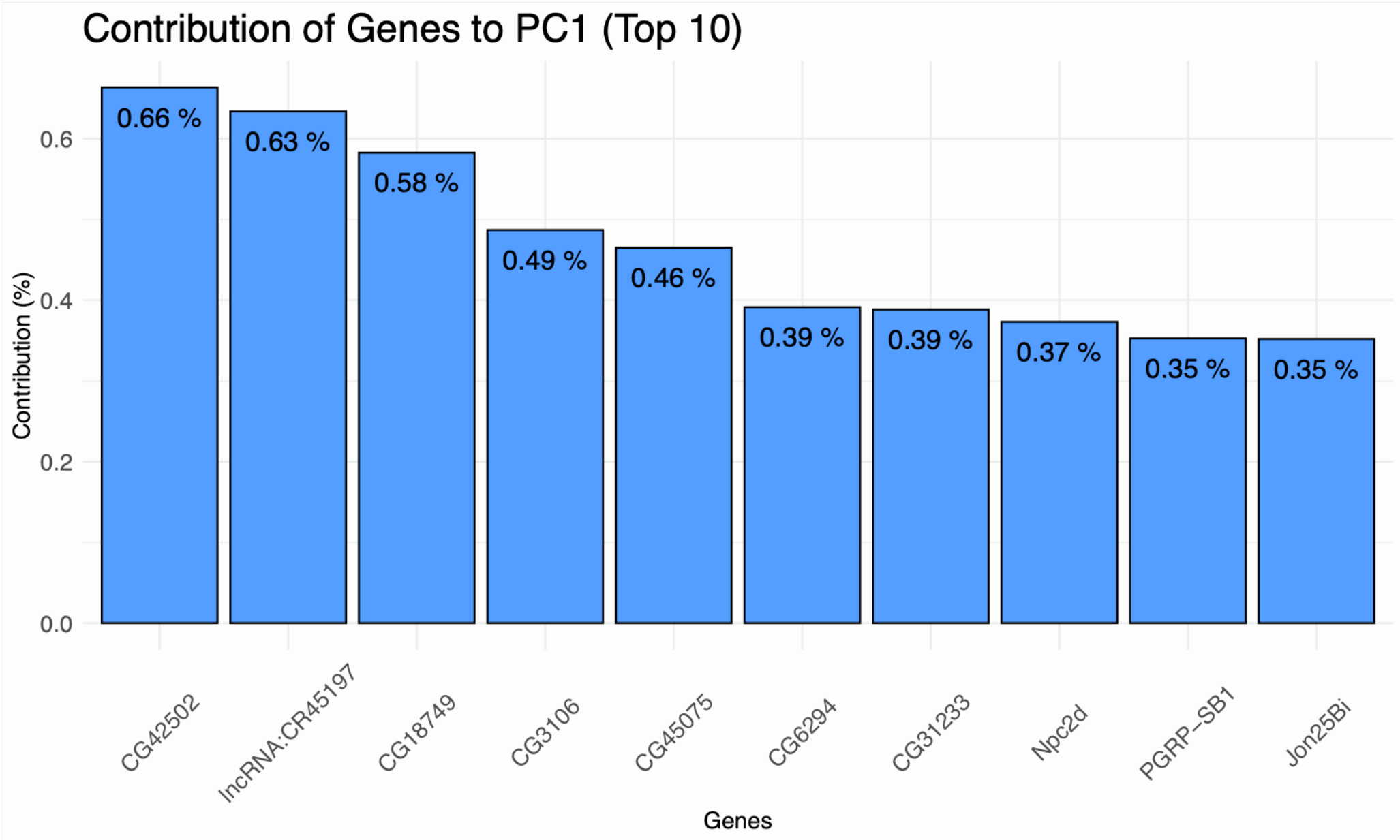

Figure S2. Gene contributions to PC1 in the RNA-seq PCA. Related to Figure 3.

The top 10 genes contributing to PC1 are shown. Bars indicate the percentage contribution of each gene to PC1. PC1 primarily separated sensitive and insensitive flies in the PCA shown in Figure 3A.
